# Cerebral Autoregulation in Unilateral Carotid Agenesis: How Low Can We Go?

**DOI:** 10.7759/cureus.24232

**Published:** 2022-04-18

**Authors:** Joana Almeida, Francisco Seixas, Carlos Mexedo, Humberto Machado

**Affiliations:** 1 Anesthesiology, Centro Hospitalar e Universitário do Porto, Porto, PRT

**Keywords:** near-infrared spectroscopy (nirs), systemic hypertension, carotid agenesis, brain perfusion, cerebral oxygenation

## Abstract

Dysgenesis of the internal carotid artery (ICA) is a rare vascular disorder. It has a variety of different grades (agenesis, aplasia, and hypoplasia) and is more common on the right side. Although the ICA is an important vessel, most patients are asymptomatic due to collateral circulation. Recognition of this rare anomaly is important, particularly when considering patients for surgeries that demand permissive hypotension. We present and discuss the perioperative implications of a rare case of congenital absence of left carotid artery proposed for an urgent laryngeal biopsy and tracheostomy. The internal jugular vein was invaded with a tumor and so was removed, affecting venous drainage.

## Introduction

Aplasia, hypoplasia, and agenesis of the internal carotid artery (ICA) are rare congenital anomalies first described by Tode in 1787 [1]. Since then, there have been less than 100 cases reported worldwide. While most patients with dysgenesis of the ICA are asymptomatic, oxygen delivery to the brain might be compromised during the perioperative period [1-3].

An estimated 1.13 billion people worldwide have hypertension. The evidence shows that patients with chronic hypertension have a right shift in cerebral blood flow (CBF) autoregulation curve. Consequently, these patients may need different cerebral perfusion pressure (CPP) targets to maintain an adequate cerebral blood flow [4,5].

Permissive hypotension represents one of the most important challenges to anesthesiologists in patients submitted to head and neck surgery. Specific positions may also jeopardize brain perfusion, e.g., neck hyperextension via the collapse of the vertebrobasilar system.

The maintenance of adequate oxygen delivery to tissues and organs, especially the brain, is a fundamental objective of the anesthetic process. Despite the devastating effects of cerebral hypoxia, the brain remains one of the least monitored organs during anesthesia [6].

We report a case of a patient with ICA left agenesis proposed for suspension microlaryngoscopy and tracheostomy. The patient provided written consent for the publication of this case report.

## Case presentation

**Figure 1:**
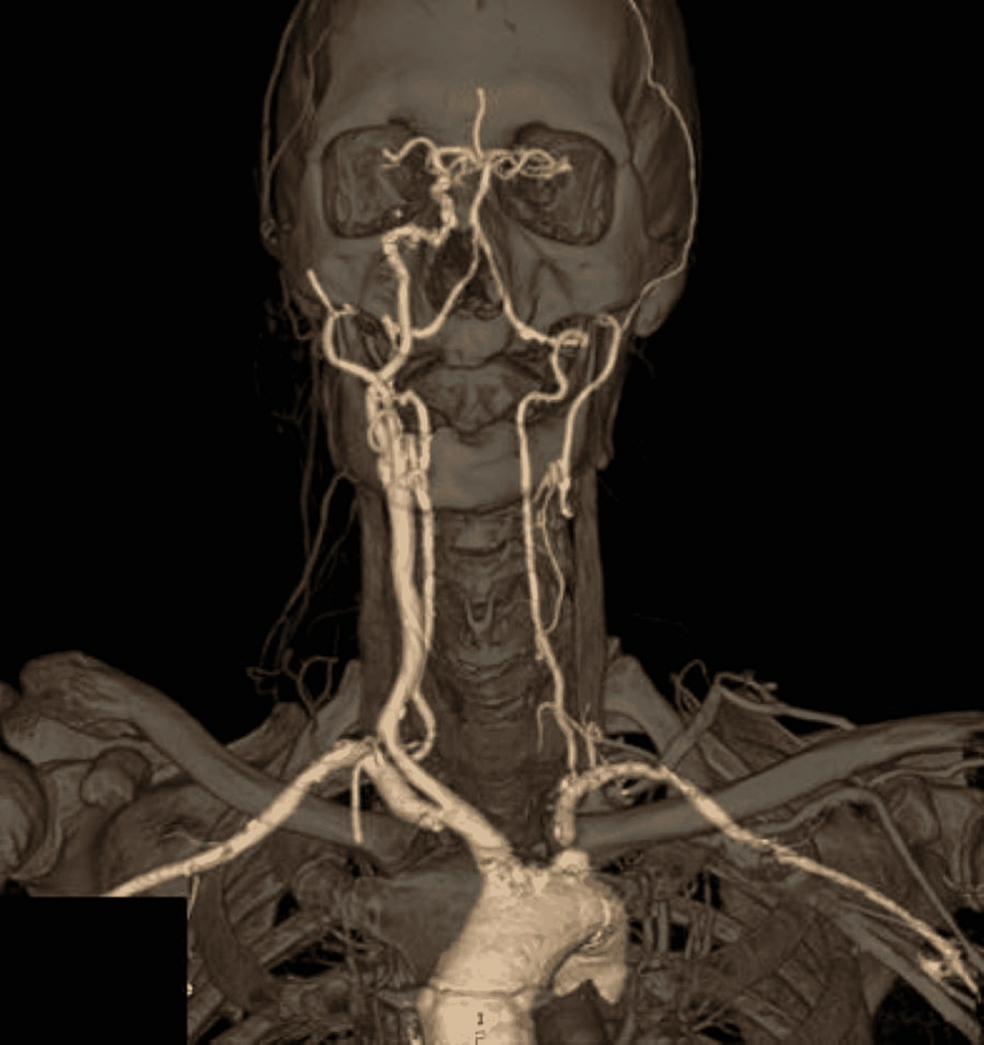
3D head and neck CTA reconstruction (anterior view).

A 58-year-old male presented for an urgent laryngeal biopsy and tracheostomy due to a glottic tumor with infra- and supraglottic extension. The patient was classified as American Society of Anesthesiologists (ASA) physical status (PS) IV and had significant comorbidities such as uncontrolled hypertension, peripheral arterial disease, type II diabetes mellitus, and active smoking. He had a previous history of a postponed nasal septoplasty due to uncontrolled hypertension (250/110 mmHg). At the time, an asymptomatic absence of the left internal carotid artery (Figure 1) was diagnosed. However, the patient did not comply with the prescribed medication and was lost follow-up.

General intravenous anesthesia was planned. We monitored the patient with five-channel electrocardiography (ECG), pulse oximetry, continuous direct arterial pressure measurement, bilateral bispectral index (BIS) monitor (BIS VISTA, Covidien, Dublin, Ireland), bilateral noninvasive cerebral oximetry (INVOS™ 5100C Cerebral/Somatic Oximeter, Covidien, Dublin, Ireland), neuromuscular function (Datex-Ohmeda's M-NMT MechanoSensor, Datex‐Ohmeda Inc., Helsinki, Finland), and end-tidal CO2. These values were automatically registered with the Centricity software (Centricity™, GE Healthcare, Chicago, IL, USA). Basal cerebral oximetry was 66R/60L, and arterial blood pressure was 223/105 mmHg (mean arterial pressure (MAP): 135 mmHg) before induction.

**Figure 2:**
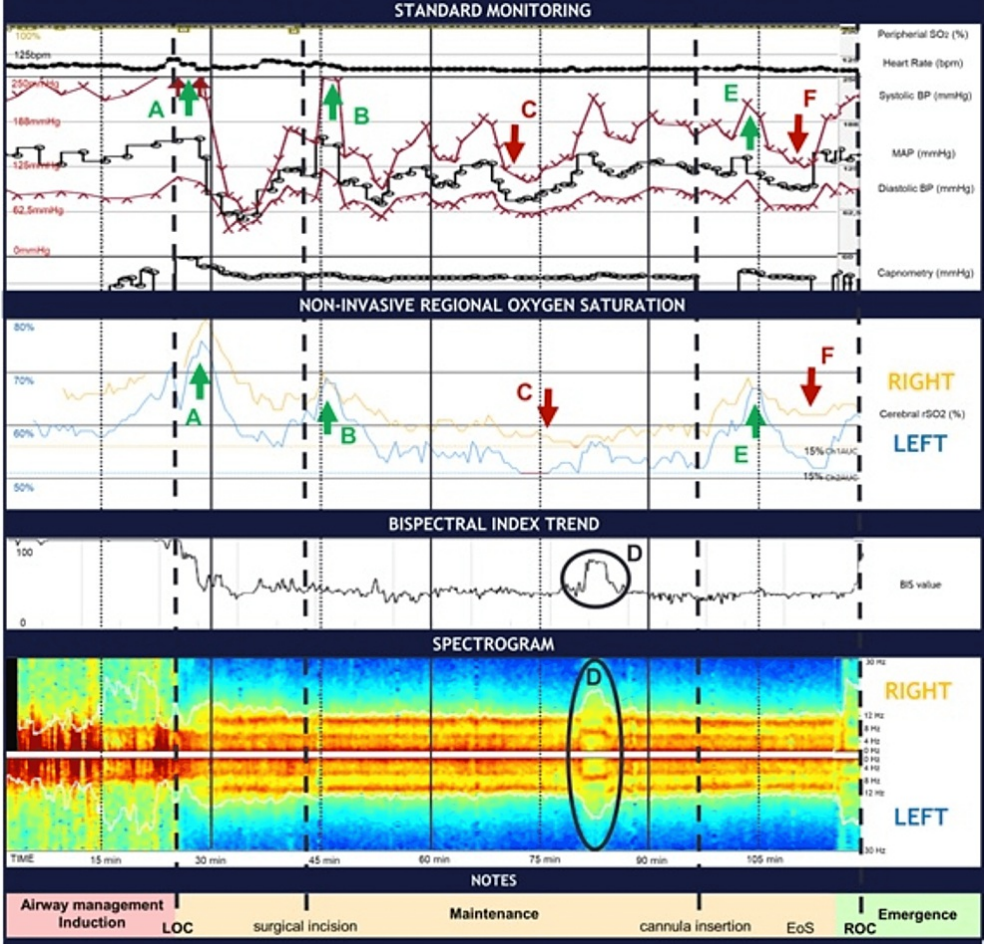
Anesthesia monitor trends from the time the patient entered the operating room to the end of the surgery, including the color density spectral array (DSA). Red arrows and letters (C and F) identify two points of MAP decrease followed by a significant increase in rSO2 asymmetry. At C (~75 minutes), MAP decreases from 125 to 78 mmHg, followed by a decrease of 15% on both rSO2. Green arrows and letters identify three hypertensive peaks (A, after intubation; B, after surgical incision; and E, after tracheal cannulation). Hemodynamic changes were accompanied by a rise in both rSO2. Simultaneously, there was a significant decrease in rSO2 asymmetry, especially when compared to preoperative values. Black circles (D letters): Propofol and remifentanil Ce were decreased, followed by a loss in power on both delta and alpha spectrum and a raise in theta and beta activity. The BIS value raised from 50 to 73, and electromyography was detected.

Preoxygenation was performed (~15 minutes, Figure 2), and remifentanil TCI (Minto model) was started. Nasal neosynephrine and lidocaine were topically administered, followed by 120 mg lidocaine IV. Endotracheal intubation was performed under fibroscopy assistance, with a 5-mm microlaryngoscopy tube. Spontaneous breathing and consciousness were maintained during the airway management. A 1% propofol TCI (Schnider model) in TCI-View mode (100 mL/hour) was started until loss of consciousness (LOC) (~25 minutes, Figure 2). Propofol Ce was recorded and then titrated according to pEEG monitoring, while remifentanil Ce was titrated based on both noxious stimulus and hemodynamic parameters. Neuromuscular blockade was obtained with rocuronium IV boluses. An invasive arterial catheter was placed, and the surgical procedure (laryngeal biopsy) was started (~45 minutes, Figure 2).

During the maintenance phase (~75 minutes, Figure 2), a sudden decrease in the MAP from 122 mmHg (188/90 mmHg) to 78 mmHg (132/65 mmHg) was observed, and both regional oxygen saturation (rSO2) reached values equivalent to 85% from baseline values. Afterward, remifentanil and propofol Ce were decreased, MAP raised, and rSO2 returned to values above 85%. After this event, an increase in the BIS value from 50 to 73 with electromyographic artifacts and with a relative loss in delta and alpha power was observed (~80 minutes, Figure 2). Propofol Ce was titrated, and 20 mg rocuronium was administered. BIS values returned to 40-60, and alpha oscillation power increased.

Tracheostomy and cannulation (~100-105 minutes, Figure 2) were uneventful. Before emergence from anesthesia (~115 minutes, Figure 2), significant asymmetry was recorded on cerebral oximetry. Postoperative analgesia was obtained with 1 g paracetamol + 30 mg ketorolac. Then, 4 mg dexamethasone was given for the prevention of postoperative nausea and vomits. Neuromuscular blockade was reverted with 200 mg sugammadex IV, and propofol infusion was stopped. After regaining consciousness, spontaneous breathing was maintained through the tracheostomy, and no neurologic deficits were observed.

## Discussion

The blood supply of the brain comes from two major vascular systems: the vertebrobasilar and carotid systems. The internal carotid arteries branch to form the anterior and middle cerebral arteries. Both vertebral arteries join to form the basilar artery that runs on the surface of the pons. These two systems anastomose in the ventral surface of the brain, in an arterial ring called the circle of Willis [1-3].

Congenital anomalies of the ICA are rare, with an estimated incidence of <0.01%. They typically occur during embryonic development in the form of hypoplasia, aplasia, and agenesis. These terms are often used interchangeably in the setting of an absent or small ICA. Left-sided carotid agenesis has a reported ratio of cases of 3:1 and is mostly clinically silent due to well-developed collateral circulations from the vertebrobasilar system via the circle of Willis. Although less frequent, the contralateral internal carotid artery and ipsilateral external carotid artery can also have an important role in collateral circulation [1-3]. Figure 1 is the reconstruction image from the angiographic CT of our patient.

Beyond ICA agenesis, the patient also presented uncontrolled refractory hypertension. Studies showed that chronic hypertension has an impact on cerebral blood flow. Patients with hypertension also have increased cerebrovascular resistance and altered cerebral autoregulation compared with healthy control patients. In 1959, Lassen defined cerebral autoregulation as the intrinsic ability of the cerebral vasculature to maintain constant cerebral blood flow despite changes in CPP. Under normal circumstances, changes in arteriolar diameter regulate cerebral blood flow and drive changes in cerebrovascular resistance, following the Hagen-Poiseuille equation. CBF is dependent on CPP and inversely proportional to cerebrovascular resistance. Maintaining adequate brain perfusion is crucial during anesthesia, and the acceptable range of blood pressures should be based on the patient’s baseline [7-9].

Brain perfusion can also be affected by the patient’s position. The positioning is a shared responsibility among the whole team: anesthesiologist, surgeon, and nurses. Microlaryngeal surgery is performed in Boyce-Jackson position using a combination of cervical flexion and atlanto-occipital extension. Some studies tried to make a connection between cervical hyperextension and rotation with reduced cerebral flow. In 1995, Weintraub et al. found that hyperextension significantly reduces flow in the vertebral artery. This hypoperfusion can lead to occlusion, suggesting that it could be an independent and modifiable risk factor for stroke. There are some case reports describing acute carotid dissection associated with neck hyperextension in a beauty salon. So, this position can be itself a reason for neurologic events in susceptible patients [10].

In this case, we advocated MAP values > 100 mmHg based on regional cerebral oxygen saturation (rScO2) to ensure adequate cerebral perfusion pressure. Postoperatively, he remained calm, with a hypertensive profile similar to his previous. Neurologic deficits were not detected. The positive outcome may be due to the adjustment of the blood pressure targets in relation to the patient’s baseline values.

## Conclusions

This case describes the interaction between cerebral perfusion and hemodynamics, and the awareness anesthesiologists should have. Highlighting the importance of moving away from standard values of MAP to assure the maintenance of cerebral autoregulation, this case provides an example of a rare vascular anomaly in a high-risk patient with significant anesthetic implications and the need to adapt and adjust hemodynamic goals.
